# Flower visitors of *Streptocarpus teitensis*: implications for conservation of a critically endangered African violet species in Kenya

**DOI:** 10.7717/peerj.10473

**Published:** 2021-01-26

**Authors:** Mark Otieno, Neelendra Joshi, Benjamin Rutschmann

**Affiliations:** 1 Department of Animal Ecology and Tropical Biology, University of Würzburg, Würzburg, Germany; 2 Department of Agricultural Resource Management, University of Embu, Embu, Kenya; 3 Department of Entomology and Plant Pathology, University of Arkansas at Fayetteville, Fayetteville, AR, USA

**Keywords:** African violets, Fruit set, Pollinator networks, Pollination, *Saintpaulia*, Taita hills

## Abstract

**Background:**

The African violets are endangered plant species restricted mainly to the Eastern Arc Mountains biodiversity hotspots in Kenya and Tanzania. These plants grow well in shaded environments with high humidity. Given their restricted geographical range and published evidence of dependance on insect vectors to facilitate sexual reproduction, understanding their pollination biology is vital for their survival.

**Methods:**

We conducted an empirical study using flower visitor observations, pan trapping and bagging experiments to establish the role of flower visitors in the fruit set of a locally endemic and critically endangered species of African violet in Taita Hills, Kenya, *Streptocarpus teitensis*.

**Results:**

The study found that fruit set is increased by 47.8% in *S. teitensis* when flowers are visited by insects. However, it is important to note the presence of putative autogamy suggesting *S. teitensis* could have a mixed breeding system involving self-pollination and cross-pollination since bagged flowers produced 26.9% fruit set.

**Conclusions:**

Insects appear to be essential flower visitors necessary for increased fruit set in *S. teitensis*. However, there is evidence of a mixed breeding system involving putative self-pollination and cross-pollination suggesting that *S. teitensis* is somewhat shielded from the negative effects of pollinator losses. Consequently, *S. teitensis* appears to be protected to a degree from the risks such as reproduction failure associated with pollinator losses by the presence of a safety net in putative self-pollination.

## Introduction

The African violets (Genus *Streptocarpus*: Gesneriaceae) are endemic plant species restricted to the Eastern Arc Mountains of south eastern Kenya and northern Tanzania (Burtt, 1958). The term “African violets” is broadly applied to species previously categorized in the genus *Saintpaulia*. This genus was promoted as a flagship taxon of East African conservation due to the highly-threatened nature of the species under it (Eastwood et al., 1998; Christenhusz, 2012), and widespread use as potted plants (Watkins, Kolehmainen & Schulman, 2002). Before the taxonomic changes made by Christenhusz (2012), only six species were recognized in *Saintpaulia*, the rest treated as subspecies or synonyms (Darbyshire, 2006; Christenhusz, 2012). Recent studies have found close relationships between African violets and other African Gesneriad genera, especially to subgenus *Streptocarpella* within the genus *Streptocarpus*. As a result, botanists have incorporated *Saintpaulia* within this subgenus. Therefore, all the species previously known as *Saintpaulia* are now referred to as *Streptocarpus* (Christenhusz, 2012; Nishii et al., 2015).

Globally, these plants are of high-value and importance in horticulture. African violets are well-known house plants and are easily cultivated from cuttings (Christenhusz, 2012). This asexual propagation (also common in the wild) has probably contributed to populations looking identical in certain areas that are geographically isolated due to topography (Darbyshire, 2006; Christenhusz, 2012). However, some species, for example, *Streptocarpus ionanthus subsp*. *grotei*, form long stolons which are very likely more significant for clonal appearance than accidental leaf cuttings. The African violets are mainly grown as indoor ornamental plants in most of Europe, North America and Australia and have been bred to create highly priced hybrids (Simiyu et al., 1996; Eastwood et al., 1998). Because of their specific ecological requirements, the wild species of the African violets (e.g., *Streptocarpus teitensis*) are valuable ecological indicators for intact ecosystems that depict a healthy habitat and plant community. The existence of endangered African violet species (e.g., *Streptocarpus teitensis* (B.L. Burtt) Christenh. (formally *Saintpaulia teitensis*)) has increasingly been threatened with habitat destruction since the late nineteenth century (IUCN SSC East African Plants Red List Authority, 2014). *Streptocarpus teitensis* is now almost extinct in the wild and usually associated with isolated and fragmented forest habitats (Kolehmainen & Mutikainen, 2006; Martins, 2008).

In Kenya, *S. teitensis* is found in Taita Hills, restricted to the Mbololo forest, the largest contiguous forest area in the Taita Hills massif (Bennun & Njoroge, 1999). These hills are home to several other endemic species, including some species of butterflies, reptiles, birds, and amphibians (Simiyu et al., 1996). Despite losing about 98% of forest cover in the last 200 years (Newmark, 1998), the Mbololo forest still holds the endemic and Critically Endangered Taita apalis (*Apalis fuscigularis*) and Taita thrush (*Turdus helleri*) (Borghesio et al., 2010), which makes the forest so important. The moisture accumulated on the slopes of these hills provides municipal water to communities within the Taita Hills and the surrounding settlements and portions of Tsavo National Park.

Wild populations of the African violets grow as patches on well-drained surfaces in shaded areas with high humidity, typically near soil free environments offered by rock surfaces (Faden, Beentje & Nyakundi, 1988; Darbyshire, 2006; Martins, 2008). The present day habitat is characterized by a highly fragmented and heterogeneous agricultural landscape interspersed with forest patches. The forest fragments where the African violets grow border crops grown in different landscape settings ranging from surroundings with low forest cover to places almost entirely enclosed by forest. Although this plant species falls within the Taita Hills Wildlife Sanctuary (Protected Planet, 2020), this does not translate into on-the-ground protection of this natural resource as the site of its occurrence is accessible unimpeded to anyone at any time.

Studies on the breeding system of the African violets have established that they are insect-pollinated (Kolehmainen & Mutikainen, 2006; Martins, 2008). However, we have only limited knowledge of which insects are effective pollinators. The plants’ floral morphology indicates a pollen-reward pollination mechanism, given the conspicuous anthers exsert beyond the short corolla tube. Given that the large yellow anthers of *Streptocarpus* dehisce in slits and the absence of nectaries led to suggestions of buzz pollination (Harrison, Möller & Cronk, 1999), which was ultimately confirmed by Martins (2008). Buzz pollination is a specialized form of pollination used mainly by some species of bees (e.g., *Amegilla* spp., *Anthophora* spp., *Xylocopa* spp. etc.) to free the pollen firmly held by the anthers (De Luca & Vallejo-Marín, 2013).

African violet species have very restricted range distributions; growing in highly fragmented habitats, and dependance on insect vectors to pollinate its flowers. Therefore, understanding its pollination biology and reproductive success is important for its conservation and propagation. A previous study documented bees, especially digger or Anthophorid bees (*Amegilla* spp.), to visit the flowers (Martins, 2008). However, little is known of how the relationship between pollinator visitation and pollination affects how much fruit set occurs. For instance, Kolehmainen & Mutikainen (2006) reported on visitors and the absence of selfing in three closely related species (*S. confusa, S. difficilis*, and *S. grotei*, all now *S. ionanthus* subsp. *grotei*; see Nishii et al., 2015). Martins (2008) reported on visitors and their behavior and comprehensively examined the buzz pollination process in *S. teitensis* by *Amegilla* bees. This study determined that once a bee lands on a flower, it grasps it with its legs, holds part of the flower in its mandibles and vibrates its wing muscles. This shaking transfers the high-frequency vibrations to the flower, causing pollen to be released from the anthers that coher at the tip by entangled hairs (Weberling, 1989). As the bee continues with shaking while in contact with the flower, it makes circular movements, which helps with adhesion of pollen on its body hairs. The bee then rises above the flower by hovering into the air while combing the pollen into the pollen baskets. The bee will then either return to another flower for another round of vibrations, effecting pollination in the process, or fly away. The term “buzz pollination” (Proctor, Yeo & Lack, 1996) stems from the sound caused by the vibrations the bee makes during the process as it holds tightly onto the flower.

We conducted an empirical study to establish the role of insect flower visitors in the reproductive success of the endemic species of African violet *S. teitensis* in order to propose measures of promoting its conservation by preserving its flower visitors. Specifically, the study aimed at: (i) establishing the relationship between flower visitors and fruit set, and (ii) developing a network of other flowering plants visited by the flower visitors of *S. teitensis*. In this study, we use flower visitors as a proxy for pollinators. Previous studies have established that dominant animal taxa (e.g., bees) visiting flowers often provide the majority of the pollination service for many plant species (Alves-dos-Santos et al., 2016). Besides, visitation networks are an excellent proxy for the pollination effectiveness of these regularly interacting species (Vázquez, Morris & Jordano, 2005). In the present study, we did not measure actual pollination, that is, pollen removal from the anthers, transportation, and deposition onto stigmas, by any of the flower-visiting species. We, therefore, use the term “flower visitors” from here onwards to refer to an essential community of insects visiting the flowers of *S. teitensis*, potentially providing the pollination service in the context described above.

## Materials and Methods

### Study region

**Figure 1:**
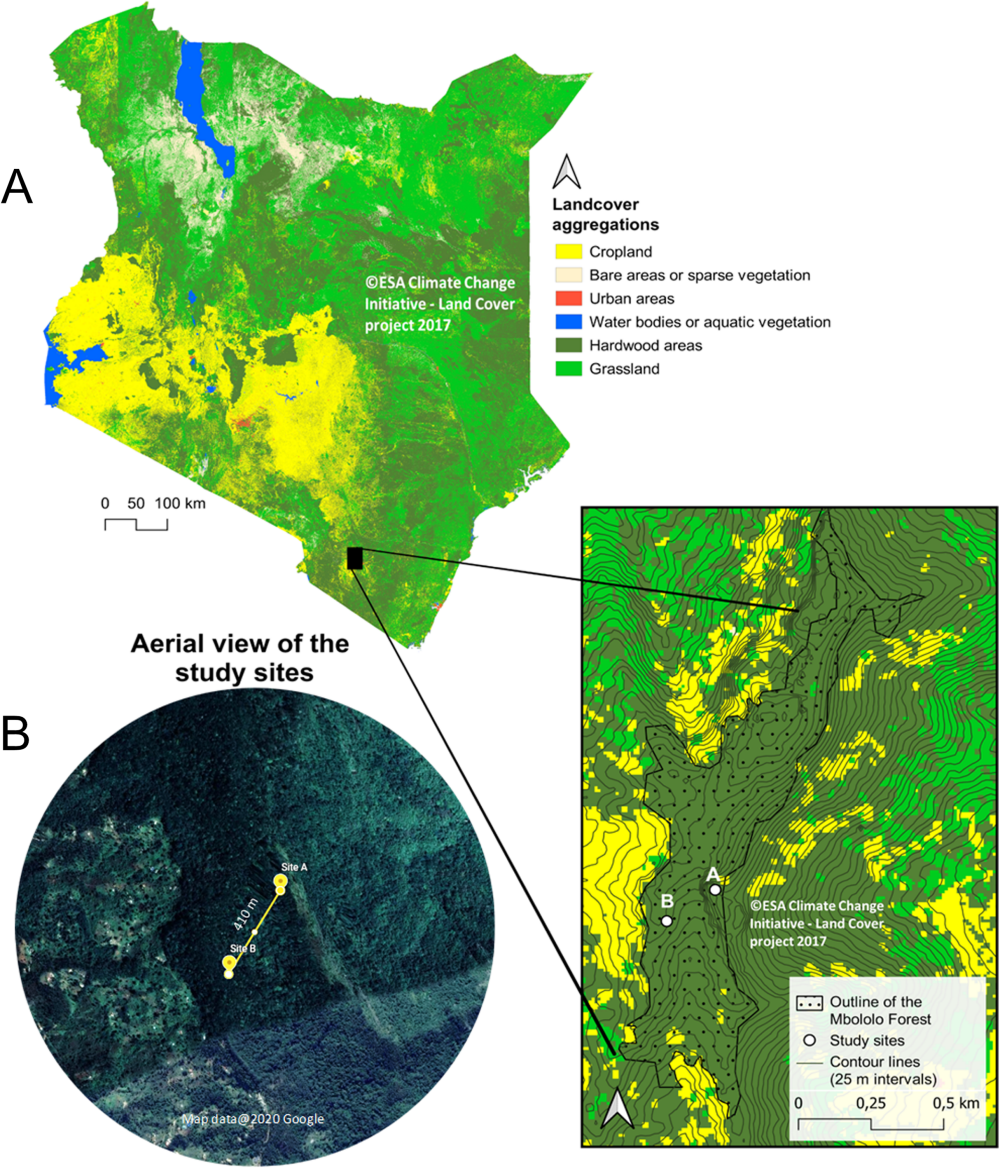
Map of Kenya showing Mbololo Forest and the study sites. (A) Land cover and forest cover data obtained from the S2 prototype LC 20m map of Africa 2016 (© Contains modified Copernicus data (2015/2016), © ESA Climate Change Initiative—Land Cover project 2017), clipped with GDAM (Global Administrative Areas) data set. Contour lines from NASA SRTM data. (B) Satellite images obtained from Google Earth (imagery courtesy of DigitalGlobe, CNES/Airbus and the U.S. Dept. of State Geographer Data via Google Earth), map data ©2020 Google.

The study was conducted in Mbololo Forest, part of the south eastern Kenya biodiversity hotspots in Taita Hills (Taita Taveta County) in Kenya at Latitude −3.295 and Longitude 38.461 (Fig. 1). The research was authorized by the National Commission for Science, Technology, and Innovation (NACOSTI)—research permit no.: NACOSTI/P/19/57668/28371 and the Kenya Forest Service (KFS)—authorization letter REF: KFS/TT/8/16/Vol.II/86. The Mbololo Forest has a high species richness as a large number of endemic plant and animal species are found here, but this region experiences threat from human-induced factors (Simiyu et al., 1996). The Taita African violet (*Streptocarpus teitensis*) is endemic to this forest and one of the Red Listed species classified as Critically Endangered by the International Union for Conservation of Nature (criteria B1ab(iii)+2ab(iii)) (IUCN SSC East African Plants Red List Authority, 2014). The growth range of *Streptocarpus teitensis* is restricted to between 1,400 and 1,850 m above sea level.

The local Taita community lives adjacent to this forest and usually grows different crops (such as native fruits and vegetables) along the hills’ steep slopes. It is common for individuals to own parcels of land at different elevations in the hilly area to obtain rainfall at different zones and thus safeguard against crop failure. This practice has resulted in intense forest clearing and an extreme fragmentation pattern of land ownership (Martins, 2008).

### Sampling design

The study area was initially surveyed from 12th to 15th February 2019 to search and map the locations of the study plant species using a systematic transect approach. Recent maps of the study area were obtained from Google Maps (Google©2019) and were used to demarcate the zones into manageable search sizes. A total of three zones of almost equal sizes (9 km^2^) were demarcated. In each of the three zones, ten 500 m transects were laid and walked by two people searching for the *S. teitensis* on either side of the transect. When the species was encountered, its location was marked using a GPS (Garmin eTrex 30) and its photograph together with the surrounding habitat taken. In order not to miss the plants using this approach, local members of the community through Community Forest Associations in Woghonyi and Mwambirwa villages closest to the forest, were asked to guide the team to known locations of the plant species. Through a combination of both approaches, these plants were found at two locations; at site A (Latitude −3.332, Longitude 38.451) and site B (Latitude −3.334, Longitude 38.448). Site A was ~6,000 m^2^ (~120 m × ~50 m) with approximately 2,300 mature plants and site B ~1,050 m^2^ (~105 m × ~10 m) with ~500 mature plants. These sites were close to each other, ~410 m apart. The plants grew either attached to rock surfaces or on bryophyte mats on the ground of the forest floor or attached to tree trunks (Appendix 1). Only the plants growing on rocks and on the ground were studied, leaving the ones on tree trunks because they were located relatively higher (up to 15 m off the ground), impeding accurate observations from the ground (Appendix 1). The proportion of these epiphytic plants was very low (less than 1% of all the plants), and their omission was unlikely to have affeced the outcome of the study.

### Visitor abundance and visitation of *S. teitensis* flowers

Insect sampling was conducted at each of the two sites to determine the species richness and abundance of flower visitors. Insects were collected using sweep nets and pan traps. At each site, one 100 m transect was laid. Because of the very rare occurrence and low abundance of *S. teitensis*, it was not possible to lay more than one transect or extend its length at each site. Sampling with pan traps along the line transects and flower visitor observations were done on diverse dates from 27th April to 2nd August 2019 for a total of 23 days. Line transects were chosen because they are useful for illustrating linear patterns along which communities of arthropods change and allow efficient sampling of many sites under time constraints (Baldock et al., 2015; Gibson et al., 2011). They provide a good way of visualizing and observing the changes in insect community or activity taking place along the line more clearly.

Timed visual counts were used during each sampling event to determine insect flower visitation rates to *S. teitensis* flowers in the period from 27th April to 2nd August 2019. Timed visual counts are unbiased methods of observing insect activity based on random chance and encounter of insects visiting the target flower cluster of interest. On each transect, five plants were randomly chosen, and on each plant, a cluster of freshly opened flowers (usually at the center of each plant stalk) were continuously observed for 30 min at different intervals from 08h30 to 16h30 during peak flowering season. Plants in Site B only had flowers during the months of April and May. During each sampling event, the field research team always wore dull-colored clothing in order not to be too conspicuous to disorient flower visitors. The identification of flower visitors was done in the field whenever possible. Those insects difficult to identify in the field were caught using sweep nets and taken to the National Museums of Kenya for identification using taxonomic identification keys. Morphospecies were used for those specimens difficult to identify using both approaches.

Pan traps were also used for sampling the relevant taxa of the general insect flower-visiting fauna within the study area (Nuttman et al., 2011). A cluster of 3 UV bright blue, yellow, and white pan traps were placed at 5 locations at intervals of 20 m along the 100 m transect. The pans were half-filled with unscented soapy water and left out for 24 h before insects trapped were collected and preserved in 75% ethanol for later identification at the National Museums of Kenya. Pan trapping was used because it is an excellent method of capturing flying arthropods responding to colored cues. Blue, yellow, and white colored bowls are used to catch different groups of insects as different insects respond to different colored cues and intensity of light reflections from these traps (Joshi et al., 2015).

### Fruit set experimental protocol

The breeding system of *S. teitensis* was assessed through flower visitor exclusion experiments to determine the proportion of fruit set attributable to these flower visitors. This was done using paired comparisons of *S. teitensis* either open or closed to flower visitors (Kolehmainen & Mutikainen, 2006; Martins, 2008). Five pairs of plants were selected along the 100 m transect in site A and two pairs in Site B. On each experimental plant, we counted all the flowers and placed a Tulle bag (fine cloth-based netting—Operandi^®^ paint strainer bags, 18.9 l, 200 µ, nylon mesh) on one plant per pair in both sites totaling to five bagged plants in site A and two in site B. We left open the second plant per pair in each site to act as controls that were open to flower visitors to assess natural pollination. The plants in each pair were close to each other (within five meters). In site B, fewer plants fitted the desired attributes for use in the pollinator exclusion experiments. The attributes were; same approximate height and breadth, same (or nearly equal) number of flowers and plants with fresh flower buds. This is why, for Site B, the number of plants is only four compared to ten in site A. The bagging experiments did not exclude only insects, but other possible non-insect faunal pollination vectors apart from wind and possibly thrips (Eliyahu et al., 2015). However, this is a standard method used in pollination ecology typical for testing self-compatibility, autogamy (Sun & Ritland, 1998; Suetsugu, 2013), and the presence of apomixis (Dupont, 2002). Specifically, the bagging method determines presumed plant pollination by excluding a specific group of visitors from accessing and, potentially pollinating the flowers.

A total of 262 flowers were marked on the 14 individual plants in both sites from April to August 2019. One hundred thirty-three (133) of these were bagged on the first set of seven plants on the pairs described above, and 129 marked on the next set of seven plants used as controls and left open to natural pollination (Appendix 2). At the end of the experiment, we counted the number of fruits formed on both the bagged and open-pollinated plants. We then quantified the amount of pollination due to insects following the formula by Ricketts et al. (2008), that is, *Insect Pollination = Open pollination [control]—Self-pollination [Tulle bags]*. In the data analysis, the fruit set attributable to flower visitors was quantified as the percentage of the difference between open and excluded flower visitors (Otieno et al., 2015).

### Flower visitor networks of *S. teitensis* and other wild flowering plants

Insects visiting the flowers of *S. teitensis* were followed whenever possible to determine which other flowers they visited within the vicinity of the sampled plants. Only the plant species that shared flower visitors with *S. teitensis* were recorded. Photos and vouchers of these plants were taken for identification by botanists. The vouchers of wild plant species with flowers were collected using standard plant material collection methods and pressed using plant presses and transported back to the laboratory for identification. Those specimen challenging to identify at the lab using standard keys and photo comparisons were taken to the National Museums of Kenya for verification from botanists. These data were later used to generate networks and species strengths of flower visitors as described in the data analysis section.

### Statistical analyses

Data were pooled for the entire sampling period (April–August 2019) and summarized to visualize general trends. Further analyses were performed in R statistical software version 3.6.1 (R Core Team, 2019). Flower visitation data were summarized to produce flower visitor species richness, which were all the insect species recorded visiting the flowers. Descriptive statistics were used to summarize flower visitation abundance data. A paired sample *t*-test was used to determine the difference between the abundance of insects caught by the pan traps in the two sites. The difference in fruit set when flowers were open to flower visitors and thus naturally pollinated or self-pollinated (from the bagged experiments) was determined using the Mann–Whitney–Wilcoxon Test since these data were not normally distributed hence failed to satisfy the assumptions of the parametric *t*-test.

Flower visitor networks were constructed to determine plant-flower visitor linkages using the *plotweb* function of the Bipartite package in R with wild plant species linked to flower visitor taxa (Dormann, 2011; Willcox et al., 2019). We focused on metrics that were known to be robust against variations in sampling effort, network size, the total number of interactions, and high proportions of singleton observations (Willcox et al., 2019). The networks were exported as pdfs to a graphics program (GIMP version 2.10.12), where they were smoothened at 300 dpi. Species strengths in these networks were calculated for each flower visitor to determine its importance in the network. Using the same data frame as for *plotweb* function that produced the networks, species strengths were obtained by running the script *“splevel” <- specieslevel(dataframe, level = “higher)”* on the model to determine whether individual insect flower visitor taxa were more important than that expected given random interactions (Willcox et al., 2019).

## Results

### Flower visitor abundance

A total of 270 flower visits to *S. teitensis* were recorded from 33 different known species/morphospecies of insects (including bees, flies, and butterflies) and some unknown insect species throughout the sampling period from 27th April to 2nd August 2019 (Table 1). The most abundant flower visitors were *Xylocopa flavorufa* (10.7%), *Anthophora conspicua* (10.3%), and *Apis mellifera* (10.0%). Some flower visitors in Table 1 appear only as morphospecies. The labeling as “morphospecies” was carefully applied based on morphological differences between related species.

**Table 1:** Different insects observed visiting the flowers of *S. teitensis*. Different insect species and morphospecies observed visiting the flowers of *S. teitensis* during 30-min timed visual observations conducted between 27th April and 2nd August 2019.

SN	Species/taxonomic group	Number of visits	% of total visits (*N* = 270)	SN	Species/taxonomic group	Number of visits	% of total visits (*N* = 270)
1	*Anthophora acraensis*	3	1.1	18	*Helina coniformis*	2	0.7
2	*Anthophora conspicua*	28	10.4	19	*Lucilia cuprina*	9	3.3
3	*Anthophora cornuta*	1	0.4	20	*Megachile basalis*	5	1.9
4	*Anthophora piligera*	3	1.1	21	*Megachile cincta*	6	2.2
5	*Anthophora sp*.	1	0.4	22	*Megachile felina*	1	0.4
6	*Anthophora torrida*	10	3.7	23	*Megachile sp*.	8	3.0
7	*Apis mellifera*	27	10.0	24	*Philoliche sagittaria*	1	0.4
8	*Apotmetus vansomereni*	6	2.2	25	*Phytomia bulligera*	5	1.9
9	*Belonogaster juncea*	1	0.4	26	*Phytomia incisa*	17	6.3
10	*Bombylius mollis*	14	5.2	27	*Rhingia trivittata*	2	0.7
11	Butterfly	3	1.1	28	*Synagris analis*	4	1.5
12	*Cathimeris sp*.	10	3.7	29	*Xylocopa flavorufa*	29	10.7
13	*Ceratina sp*.	9	3.3	30	*Xylocopa hottentotta*	1	0.4
14	*Chrysomya regalis*	3	1.1	31	*Xylocopa modesta*	7	2.6
15	*Congomochtherus sp*.	1	0.4	32	*Xylocopa nigrita*	17	6.3
16	*Dichaetomyia pilifemur*	4	1.5	33	*Xylocopa sp*.	3	1.1
17	*Geron sp*.	10	3.7	34	Unidentified	19	7.0

**Note:**

SN, Serial Number.

**Figure 2:**
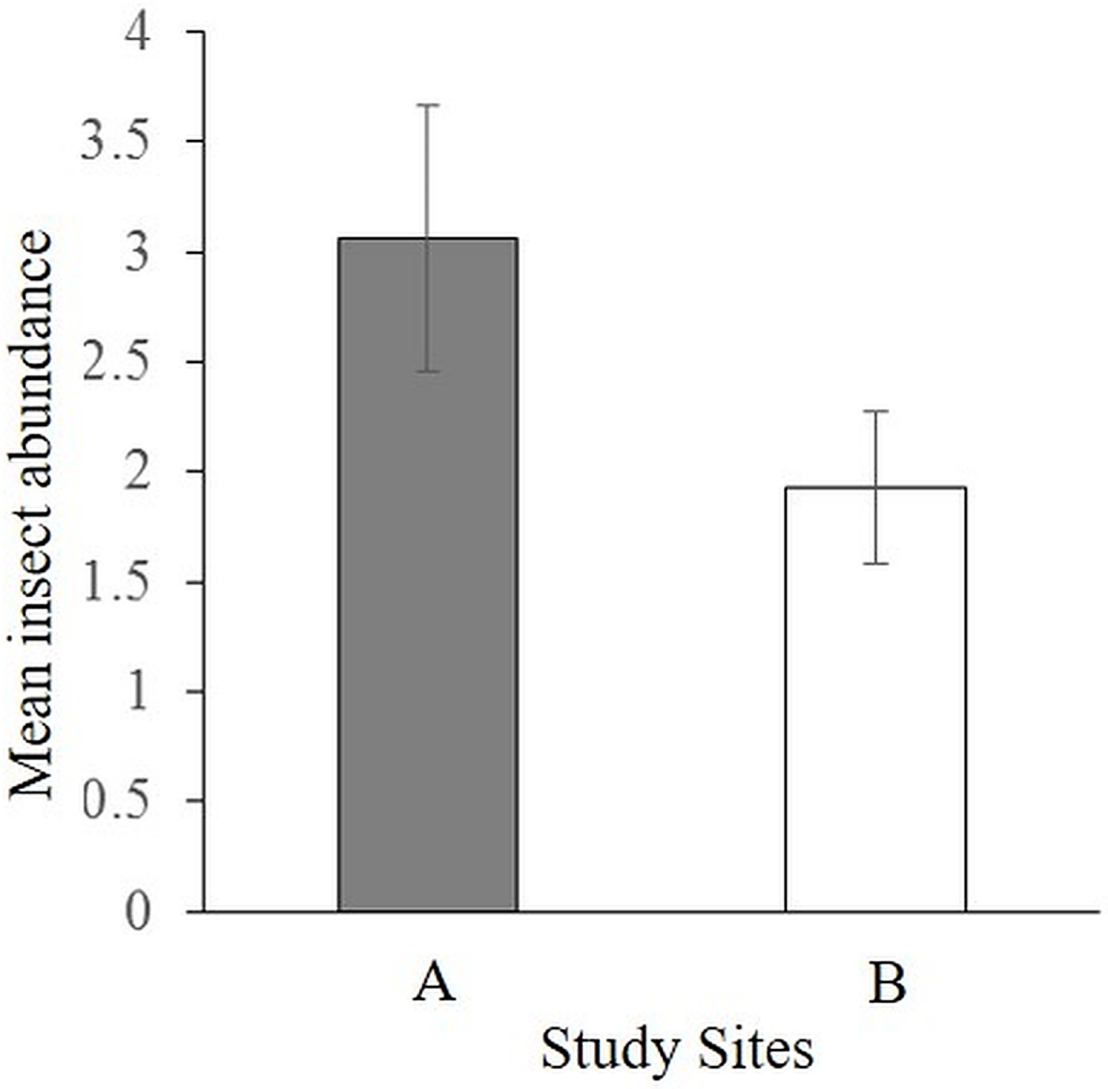
Mean abundance of insects caught by all pan traps per site. Insects caught in yellow, blue, and white pan traps along 100 m line transects in sites A and B on diverse dates between 27th April and 2nd August 2019 during the peak flowering season of *S. teitensis*.

The pan traps caught 75 insects, mostly dominated by flies (Diptera; Appendix 3) with significantly more insects caught in site A (46 individuals) than site B (29 individuals) (*t* = 1.6, df = 28, *P* = 0.05) (Fig. 2). There was no significant difference between the abundance of insects caught in the three different colored pan traps (*F*_2,27_ = 0.4, *P* = 0.68). It can be reasonably considered that insects trapped in the blue pans were more likely to be of relevance to African violet visitation than those trapped in the yellow and white pans since *S. teitensis* flowers are blue-mauve. However, where more than a few insects were caught, there did not seem to be a color preference (see Appendix 3).

#### Streptocarpus teitensis fruit set

**Figure 3:**
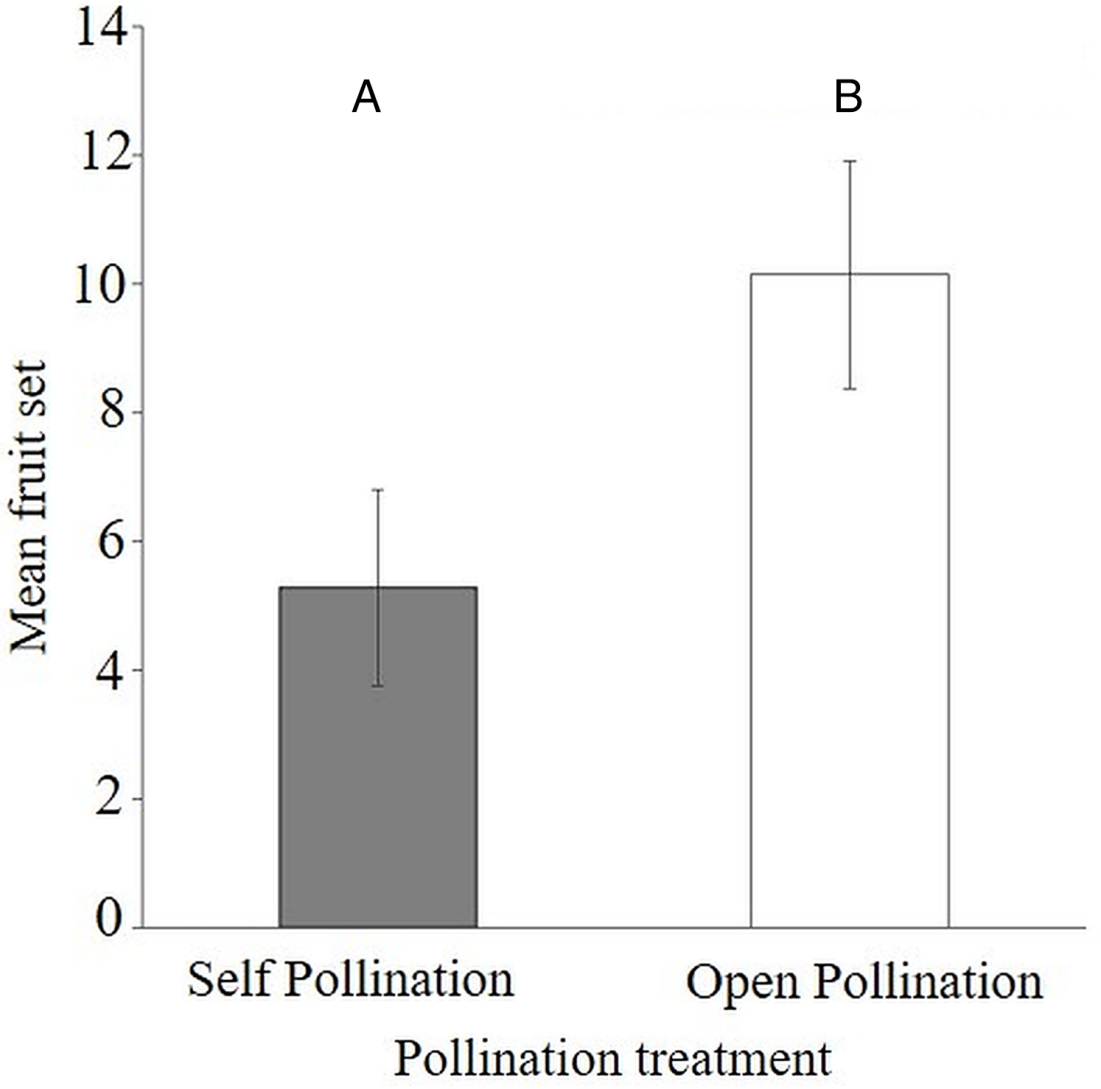
Comparisons of fruit set per plant between bagged treatment and plants under natural pollination. Fruit set of *S. teitensis* under (A) self pollination and (B) open pollination on plant pairs selected along 100 m line transects in sites A and B between April and August 2019.

The results of the flower visitor exclusion experiment showed that there was better fruit set under natural pollination (54.5 ± 9.1%) compared to bagged flowers (26.9 ± 7.4%) (Appendix 4). This difference was statistically significant as the average fruit set was reduced (Mann–Whitney–Wilcoxon Test; *W* = 40.5, *P* < 0.047) when visitors were excluded from the flowers of *S. teitensis* (Mean = 5.3 ± 1.5 S.E.) compared to naturally pollinated flowers (Mean 10.1 ± 1.8 S.E.) (Fig. 3). In the bagging experiment, 37 fruits formed from the 133 bagged flowers. These figures represent a 0.28 fruit to flower ratio compared to a ratio of 0.55 under natural pollination (71 fruits developed from the 129 marked flowers; Appendix 4). The difference between fruit set on naturally-pollinated and bagged plants based on the formula by Ricketts et al. (2008) was 4.9 fruits derived from 10.1 (mean fruit set under natural pollination) subtracted from 5.3 (mean fruit set in bagged flowers). Insect pollination, therefore, accounted for 47.8% fruit set.

### Flower visitor networks

**Figure 4:**
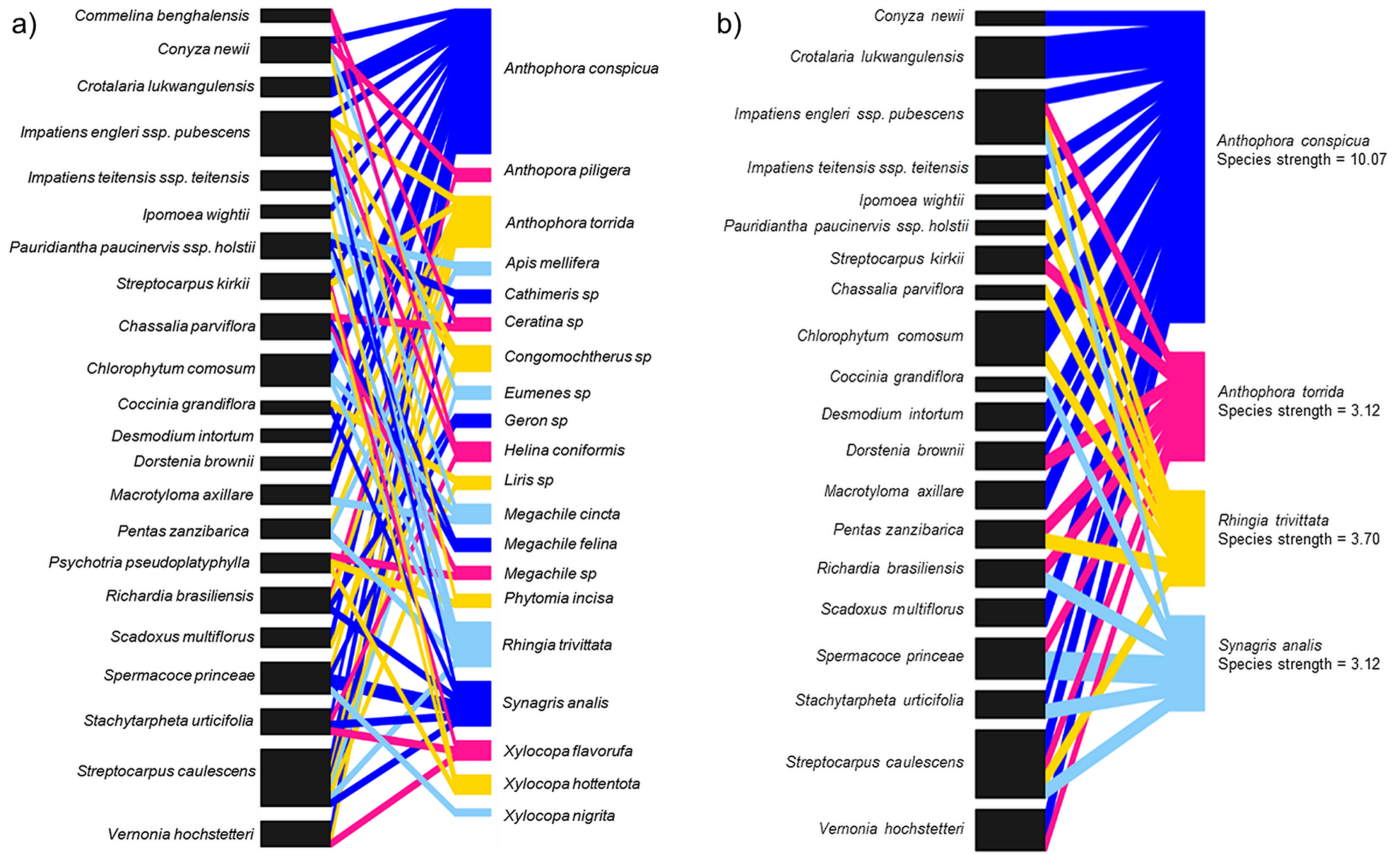
Insect flower visitor networks of shared species between *S. teitensis* and other wild flowering plants. Insect flower visitor networks of (A) all species shared between *S. teitensis* and other wild flowering plants, and (B) the four main insect species shared between *S. teitensis* and other wild flowering plants in Mbololo Forest, Kenya.

In total, 20 species of insects were shared among 22 species of flowering plants including two other *Streptocarpus* species; *Streptocarpus caulescens* and *Streptocarpus kirkii* (Fig. 4A; Appendix 5A) found in the study region. We refined this network by retaining the top four flower visitor species based on species strengths above 1.5 and reconstructed the networks (Fig. 4B; Appendix 5B). This was done to avoid a generalized network that includes insignificant flower visitors known only to steal nectar—although this is not relevant for *S. teitensis* as its flowers do not produce nectar or those that visit the flowers only by chance. The plant species visited by these four insect species were retained in the network as food sources sought by these insects in addition to *S. teitensis. Anthophora conspicua* dominated the network (species strength = 10.1), followed by *Rhingia trivittata* (species strength = 3.7), *Anthophora torrida* (species strength = 3.1), and *Synagris analis* (species strength = 3.1) (Fig. 4B).

## Discussion

### Flower visitor abundance and fruit set

This study has revealed the importance of insect flower visitors in the reproduction of *S. teitensis*, contributing nearly 50% of the fruit set. However, it is important to note the presence of putative autogamy because fruit set occurred in bagged flowers, suggesting that *S. teitensis* could be having a mixed breeding system involving self-pollination (autogamy) and cross-pollination (xenogamy) (however, see below). Other than digger bees (anthophorids and amegillas) previously reported to be the main flower visitors (Martins, 2008), flies and wasp species were also frequent flower visitors of *S. teitensis*. This finding is important because these insects add to the list of visitors to *S. teitensis* flowers, although their specific contribution to its pollination is as yet unknown.

In general, using fruit set as a predictor of reproductive success in Gesneriaceae is not straightforward. It is known that the ovaries of African violet flowers can have more than 1,000 ovules (Kolehmainen & Mutikainen, 2006), but fruit in Gesneriaceae can form with even only a few seeds developing (Wang, Michael Moeller & Cronk, 2004). Equating fruit set as reproductive success is therefore problematic. Irrespective of this, our findings on fruit set are in contrast to those by Kolehmainen & Mutikainen (2006), studying the reproductive ecology of three closely related species. Whereas our study found 26.9 ± 7.4% S.E. fruit set to occur in bagged flowers and 54.5 ± 9.1% S.E. under natural pollination, Kolehmainen & Mutikainen (2006) found no fruit set in bagged flowers, 100% in pollinated flowers, and ~60–70% fruit set under natural conditions suggesting that the species studied were pollen limited. *Streptocarpus teitensis* may also be pollen limited as it does not produce 100% fruit set under any condition, and may be similar to the other species. However, because artificial pollination was not carried out, this hypothesis needs testing. Kolehmainen & Mutikainen (2006) attributed their findings to potential resource allocation from the naturally pollinated to the hand-pollinated flowers and noted that pollinators seemed to be necessary for the sexual regeneration of *Streptocarpus* based on the finding of zero fruit set under the bagging treatment. In our study, however, the bagged experiments’ fruit set can be explained in two ways. First, *S. teitensis* was not among the species studied by Kolehmainen & Mutikainen (2006). Species differences in breeding systems do occur within the same genus, and because of this, fruit set can result from self-pollination in some species, but not others in the same genus, as observed in other *Streptocarpus* species (Hughes et al., 2005). Species of the same genus could be on different evolutionary trajectories, with some closer towards inbreeding while others having mixed reproductive systems involving selfing and cross-pollination. The latter seems at first to be supported by our data for *S. teitensis*. The second explanation is that smaller animal vectors, for example, thrips (Grice, 2013), which were not impeded by the netting, could have promoted self-pollination and possibly flew short distances to the next plants promoting cross-pollination. Thrips have been reported to facilitate self-pollination, resulting in fruit set (Eliyahu et al., 2015). However, our study did not check for the presence of thrips on flowers as these results were not anticipated. Thus, the potential of thrips as pollinators of *S. teitensis* needs to be investigated more closely to discount their contribution to fruit set and to fully understand this plant species’ breeding system. Similarly, seed set, rather than fruit set, under natural conditions, and pollen limitation, that is, shortage of pollinators, need to be investigated to obtain a clearer picture of the reproductive strategy in *S. teitensis* (Wang, Michael Moeller & Cronk, 2004). It is important also to note that this study was conducted over a single flowering season. Considerable variations between seasons can occur due to different factors such as rainfall patterns, temperatures, etc. The differences between our study and Kolehmainen & Mutikainen (2006) indicate the existence of a potentially more complex scenario with more variables at work.

In this study, pan trap data were not comparable to visual observation apart from only one genus of calliphorid flies, *Chrysomya*. Notably, no bee species were recorded in the pan traps. These results are not surprising for tropical systems. Bawa (1990) reported the paucity of bee abundance and the difficulty in sampling bees in dense forest habitats. Using pan traps to sample pollinators in a dense forest habitat, although cost-effective, can be highly selective and biased towards particular taxa (Cane, Minckley & Kervin, 2000; Droege et al., 2010). The pans had a high proportion of dipterans. These taxa are abundant in forest habitats and are mostly attracted to the colors of the pan traps. When sampling dipterans in forested or dense habitats, pan trapping is recommended for this reason (Dirrigl, 2012). For bees relevant for the pollination of *S. teitensis* (Martins, 2008), pan trapping may not be the most suitable method because bee species in forested habitats are unevenly distributed; bees and other larger-bodied insects forage primarily in the canopy, while smaller insect species in the sub-canopy trees (Bawa, 1990). Although these bees occasionally visit flowers in the sub-canopy, for example, *S. teitensis*, chances of trapping them in the pans is low due to their low abundance. This could be the reason why the pan traps did not sample bee species and were highly skewed towards dipteran species.

### Flower visitor networks

We established a network of flower visitors shared between *S. teitensis* and the species of other flowering plants including two *Streptocarpus* species; *S. caulescens* and *S. kirkii* in the study area. In this network, we found some plant species endemic in Taita Hills, for example, *Impatiens teitensis ssp. teitensis* and *Impatiens engleri ssp. pubescens*. The four flower visitors in the network presented in Fig. 4B are potential pollinators of *S. teitensis* in our study system based on the flower visitation data. In addition to the two anthophorid species in this network, *Rhingia trivittata* (Syrphidae) is also an essential flower visitor in the network and a potential pollinator. The adults of all known syrphid species are exclusive pollen and nectar feeders (Rotheray & Gilbert, 2011) although some species are known to only steal nectar from the flowers of some plant species (Branquart & Hemptinne, 2000; Lucas et al., 2018). Stealing nectar is not relevant to *S. teitensis* since they do not have nectaries but have pollen, therefore the visits by syrphids to the flowers was most likely for pollen collection. Not much information is available for *Synagris analis* (Eumenidae) regarding its pollen or nectar utilization but based on its visitation frequencies, it appears a vital flower visitor both to *S. teitensis* and in the network with other flowering plants.

The findings on flower visitors discussed above are key for the survival of the plant species as they are linked to each other via shared flower visitors. Therefore the conservation of these flowering plant species sharing flower visitors with *S. teitensis* will benefit a great deal from protecting the flower-visiting insect species. While indirect interactions between plants by shared flower visitors are often assumed competitive, sharing these visitors can be advantageous if plant species attract or maintain flower visitors and pollinator populations together (Möller, 2004). Our data suggest that the survival of the population and the coexistence of *S. teitensis* and the other flowering plant species can be encouraged by positive interactions with flower visitors leading to successful reproduction on both parts, plants and insects.

Given the threats facing *S. teitensis* and its interconnection in the network of flower visitors established in this study connecting it with the other flowering plants, the stability of this network may be at risk from local extinctions of plant species or flower visitor species and habitat change. *Streptocarpus teitensis* may, however, survive to some degree the impacts of such threats by having a putative mixed breeding system as it appears to be shielded to a degree from these risks by the presence of a safety net in putative self-pollination as indicated by our data. Additionally, its presence in the network is a better protection from pollinator loss since sharing a network of flower visitors with other flowering plants provides insurance for ecosystem stability. This aspect makes it critically important to understand better the mechanisms that drive network stability and dynamics. It is essential to ensure these flowering plant species are well managed to maintain the flower visitor community that is in return key to pollination and fruit set in the African violets and other wild flowering plant species. This management can begin with assessing the main threats to these plant species (e.g., habitat loss, herbivory, pests, etc.) and building a protection plan to mitigate these threats by involving local stakeholders (e.g., awareness building) and national agencies (e.g., formal protection).

In this study, the species strength values indicated that visitation across the network was dominated by four shared species, namely, *Anthophora conspicua*, *Anthophora torrida*, *Rhingia trivittata*, and *Synagris analis*. Previous studies have shown that *S. teitensis* benefits from pollination by anthophorid bees (Martins, 2008), the dominant flower visitors. Therefore, we can deduce that these dominant flower-visiting taxa provide most of the pollination service for the majority of the plants in the network established (Alves-dos-Santos et al., 2016). The flower visitor networks are an excellent proxy for the pollination effectiveness of regularly interacting species (Vázquez, Morris & Jordano, 2005). This means that effort should be put in place to ensure the networks’ stability. Based on the flower visitation and the pollinator network results, digger bees (Anthophorini: Apidae) appear to be important visitors for both *S. teitensis* and other flowering plants that grow within the same habitat and vicinity of the African Violet populations. To ensure continuous delivery of pollination services, this group of bees needs to be protected. This can be achieved by first understanding their ecological needs, including other food plants and nectar and pollen sources apart from *S. teitensis*, nesting resources and reproductive needs. Secondly, by protecting these resources in the local areas around *S. teitensis* populations and/or through actions that enhance these resources, for example, habitat creation or improved management. As *S. teitensis* flowers exclusively produce pollen as the reward to pollinators as they do not have a disc and thus do not produce nectar, the insects included in the network also primarily utilize pollen for their energy requirements and in that of their brood. One consequence of this dietary specialization is that *S. teitensis*, together with the other flowering plant species in the guild that primarily produce pollen reward to pollinators (e.g., *Streptocarpus caulescens* and *Streptocarpus kirkii*) stand to benefit the most from the maintenance of the suite of flower visitors shared among them.

## Conclusions

Insects appear to be essential flower visitors of *S. teitensis* necessary for increased fruit set. However, the results of bagging experiments may indicate the presence of a mixed breeding system involving putative self-pollination and cross-pollination suggesting that *S. teitensis* is somewhat shielded from negative effects of pollinator losses. The establishment of many shared flower visitors between *S. teitensis* and other wild flowering plants calls for conservation measures to safeguard these insect flower visitors to preserve their ecosystem stability. This will potentially reduce the risks such as declines in the reproduction associated with pollinator losses among pollinator dependent plant species. However, *S. teitensis* appears to be shielded to a degree from these risks by the presence of a safety net in putative self-pollination perhaps aided by thrips. Future work needs to investigate seed set, rather than fruit set, under natural conditions, and pollen limitation to obtain a clearer picture of the reproductive strategy in *S. teitensis*.

## Supplemental Information

10.7717/peerj.10473/supp-1 Supplemental Information 1 The pollinator networks code used in the Bipartite package in R to construct the flower visitor networks.

10.7717/peerj.10473/supp-2 Supplemental Information 2 African violets flower visitors dataset. Different insect species and morphospecies observed visiting the flowers of *S. teitensis* during 30-minute timed visual observations conducted between 27^th^ April and 2^nd^ August 2019

10.7717/peerj.10473/supp-3 Supplemental Information 3 African violet pantrap dataset. Insects caught in yellow, blue, and white pan traps along 100 m line transects in sites A and B on diverse dates between 27th April and 2nd August 2019 during the peak flowering season of *S. teitensis*.

10.7717/peerj.10473/supp-4 Supplemental Information 4 African violets flower visitor networks dataset. Species of insect flower visitors shared between *S. teitensis* and other wild flowering plants recorded on diverse dates between 27th April and 2nd August 2019 in Mbololo Forest, Kenya.

10.7717/peerj.10473/supp-5 Supplemental Information 5 A flowering *S. teitensis* plant attached to a tree trunk at approximately 15 m from the ground in Mbololo Forest, Kenya.

10.7717/peerj.10473/supp-6 Supplemental Information 6 Photos of bagged, unbagged flowers and fruits *S. teitensis* in Mbololo Forest, Kenya.

10.7717/peerj.10473/supp-7 Supplemental Information 7 Species caught on pan traps within the vicinity of *S. teitensis* in Mbololo Forest, Kenya. Insects caught in yellow, blue, and white pan traps along 100 m line transects in sites A and B on diverse dates between 27th April and 2nd August 2019 during the peak flowering season of *S. teitensis*.

10.7717/peerj.10473/supp-8 Supplemental Information 8 African violets fruit set data. Fruit set of *S. teitensis* under self and open pollination on five plant pairs selected along 100 m line transects in sites A and B between April and August 2019.

10.7717/peerj.10473/supp-9 Supplemental Information 9 Species strength of flower visitor networks. Species strengths of *(a) S. teitensis* flower visitors visiting other flowering plants and (b) top four *S. teitensis* flower visitors visiting other flowering plants on diverse dates between 27th April and 2nd August 2019 in Mbololo Forest, Kenya.

## Additional Information and Declarations

### Field Study Permissions

The following information was supplied relating to field study approvals (i.e., approving body and any reference numbers): The National Commission for Science, Technology and Innovation (NACOSTI) granted the permit (research permit no.: NACOSTI/P/19/57668/28371) to conduct this research and the Kenya Forest Service authorized access to Mbololo forest (authorization letter REF: KFS/TT/8/16/Vol.II/86).

### Data Availability

The following information was supplied regarding data availability: Data are available in the Appendices and Supplemental Files.

## References

[ref-1] Alves-dos-Santos I, Silva C, Pinheiro M, Kleinert AMP (2016). When a floral visitor is a pollinator?. Rodriguésia.

[ref-2] Baldock KCR, Goddard MA, Hicks DM, Kunin WE, Mitschunas N, Osgathorpe LM, Potts SG, Robertson KM, Scott AV, Stone GN, Vaughan IP, Memmott J (2015). Where is the U.K.’s pollinator biodiversity? The importance of urban areas for flower-visiting insects. Proceedings of the Royal Society B: Biological Science.

[ref-3] Bawa KS (1990). Plant-pollinator interactions in tropical rain forests. Annual Review of Ecology, Evolution, and Systematics.

[ref-4] Bennun L, Njoroge P (1999). Important bird areas in Kenya.

[ref-5] Borghesio L, Samba D, Githiru M, Bennun L, Norris K (2010). Population estimates and habitat use by the Critically Endangered Taita Apalis *Apalis fuscigularis* in south-eastern Kenya. Bird Conservation International.

[ref-6] Branquart E, Hemptinne JL (2000). Selectivity in the exploitation of floral resources by hoverflies (Diptera: Syrphinae). Ecography.

[ref-7] Burtt BL (1958). Studies in the Gesneriaceae of the Old World 15: the genus Saintpaulia. Edinburgh Journal of Botany.

[ref-8] Cane JH, Minckley RL, Kervin LJ (2000). Sampling bees (Hymenoptera: Apiformes) for pollinator community studies: pitfalls of pan-trapping. Journal of Kansas Entomological Society.

[ref-9] Christenhusz M (2012). On African violets and Cape primroses-towards a monophyletic *Streptocarpus* (Gesneriaceae). Phytotaxa.

[ref-10] Darbyshire I, Beentje HJ, Ghazanfar SA (2006). Gesneriaceae. Flora of Tropical East Africa.

[ref-11] De Luca PA, Vallejo-Marín M (2013). What’s the ‘buzz’ about? The ecology and evolutionary significance of buzz-pollination. Current Opinion in Plant Biology.

[ref-12] Dirrigl FJ (2012). Effectiveness of pan trapping as a rapid bioinventory method of freshwater shoreline insects of subtropical Texas. Southwestern Entomologist.

[ref-13] Dormann CF (2011). How to be a specialist? Quantifying specialisation in pollination networks. Network Biology.

[ref-14] Droege S, Tepedino VJ, Lebuhn G, Link W, Minckley RL, Chen Q, Conrad C (2010). Spatial patterns of bee captures in North American bowl trapping surveys. Insect Conservation and Diversity.

[ref-15] Dupont YL (2002). Evolution of apomixis as a strategy of colonization in the dioecious species Lindera glauca. (Lauraceae). Popululation Ecology.

[ref-16] Eastwood A, Bytebier B, Tye H, Tye A, Robertson A, Maunder M (1998). The conservation status of Saintpaulia. Curtis’s Botanical Magazine.

[ref-17] Eliyahu D, McCall AC, Lauck M, Trakhtenbrot A, Bronstein JL (2015). Minute pollinators: the role of thrips (Thysanoptera) as pollinators of pointleaf manzanita *Arctostaphylos pungens* (Ericaceae). Journal of Pollination Ecology.

[ref-18] Faden RB, Beentje HJ, Nyakundi DO (1988). Checklist of the forests plant species. In Beentje HJ, ed. An ecological and floristic study of the forests of the Taita hills, Kenya. Utafiti.

[ref-19] Gibson R, Knott B, Eberlein T, Memmott J (2011). Sampling method influences the structure of plant-pollinator networks. Oikos.

[ref-20] Grice M (2013). Back to Basics: Gesneriad Flower Parts. https://www.gesneriadsociety.org/wp-content/uploads/2016/05/Gleanings2013.11revised.pdf.

[ref-21] Harrison CJ, Möller M, Cronk QCB (1999). Evolution and development of floral diversity in *Streptocarpus* and *Saintpaulia*. Annals of Botany.

[ref-22] Hughes M, Möller M, Bellstedt DU, Edwards TJ, De Villiers M (2005). Refugia, dispersal and divergence in a forest archipelago: a study of *Streptocarpus* in eastern South Africa. Molecular Ecology.

[ref-23] IUCN SSC East African Plants Red List Authority (2014). Saintpaulia teitensis. IUCN Red List of Threatened Species.

[ref-24] Joshi NK, Leslie T, Rajotte EG, Kammerer MA, Otieno M, Biddinger DJ (2015). Comparative trapping efficiency to characterize bee abundance, diversity, and community composition in apple orchards. Annals of the Entomological Society of America.

[ref-25] Kolehmainen J, Mutikainen P (2006). Reproductive ecology of three endangered African violet (Saintpaulia H. Wendl.) species in the East Usambara Mountains, Tanzania. African Journal of Ecology.

[ref-26] Lucas A, Bodger O, Brosi BJ, Ford CR, Forman DW, Greig C, Hegarty M, Jones L, Neyland PJ, De Vere N (2018). Floral resource partitioning by individuals within generalized hoverfly pollination networks revealed by DNA metabarcoding. Scientific Reports.

[ref-27] Martins DJ (2008). Pollination observations of the african violet in the Taita Hills, Kenya. Journal of East African Natural History.

[ref-28] Möller DA (2004). Facilitative interactions among plants via shared pollinators. Ecology.

[ref-29] Newmark WD (1998). Forest Area, fragmentation, and loss in the eastern arc mountains: implications for the conservation of biological diversity. Journal of East African Natural History.

[ref-30] Nishii K, Hughes M, Briggs M, Haston E, Christie F, DeVilliers MJ, Hanekom T, Roos WG, Bellstedt DU, Möller M (2015). Streptocarpus redefined to include all Afro-Malagasy Gesneriaceae: molecular phylogenies prove congruent with geography and cytology and uncovers remarkable morphological homoplasies. Taxon.

[ref-31] Nuttman CV, Otieno M, Kwapong PK, Combey R, Willmer P, Potts SG (2011). The utility of aerial pan-trapping for assessing insect pollinators across vertical strata. Journal of Kansas Entomologial Society.

[ref-32] Otieno M, Sheena CS, Woodcock BA, Wilby A, Vogiatzakis IN, Mauchline AL, Gikungu MW, Potts SG (2015). Local and landscape effects on bee functional guilds in pigeon pea crops in Kenya. Journal of Insect Conservation.

[ref-33] Protected Planet (2020). Taita hills wildlife sanctuary. https://www.protectedplanet.net/taita-hills-wildlife-sanctuary.

[ref-34] Proctor M, Yeo P, Lack A (1996). The natural history of pollination.

[ref-35] R Core Team (2019). R: a language and environment for statistical computing.

[ref-36] Ricketts TH, Regetz J, Steffan-Dewenter I, Cunningham SA, Kremen C, Bogdanski A, Gemmill-Herren B, Greenleaf SS, Klein AM, Mayfield MM, Morandin LA, Ochieng’ A, Viana BF (2008). Landscape effects of on crop pollination services, are there general patterns?. Ecology Letters.

[ref-38] Rotheray GE, Gilbert F (2011). The natural history of hoverflies.

[ref-39] Simiyu SW, Muthoka P, Jefwa J, Bytebier B, Pearce TR, Van der Maesen LJG, Van der Burgt XM, Van Medenbach de Rooy JM (1996). The conservation status of the genus Saintpaulia in Kenya. The Biodiversity of African Plants. Proceedings of the XIV AETFAT Conference Wageningen. The Netherlands.

[ref-41] Suetsugu K (2013). Autogamous fruit set in a mycoheterotrophic orchid *Cyrtosia septentrionalis*. Plant Systematics and Evolution.

[ref-42] Sun M, Ritland K (1998). Mating system of yellow starthistle (*Centaurea solstitialis*), a successful colonizer in North America. Heredity.

[ref-43] Vázquez DP, Morris WF, Jordano P (2005). Interaction frequency as a surrogate for the total effect of animal mutualists on plants. Ecology Letters.

[ref-44] Wang CN, Michael Moeller M, Cronk Q (2004). Aspects of sexual failure in the reproductive process of a rare bulbiliferous plant, Titanotrichum oldhamii (Gesneriaceae), in subtropical Asia. Sexual Plant Reproduction.

[ref-48] Watkins C, Kolehmainen J, Schulman L (2002). The wild African violet Saintpaulia (Gesneriaceae)—an interim guide.

[ref-46] Weberling F (1989). Morphology of flowers and inflorescences. Nordic Journal of Botany.

[ref-47] Willcox BK, Howlett BG, Robson AJ, Cutting B, Evans L, Jesson L, Kirkland L, Jean-Meyzonnier M, Potdevin V, Saunders ME, Rader R (2019). Evaluating the taxa that provide shared pollination services across multiple crops and regions. Scientific Reports.

