# Supplementary figures and images for: Flower visitors of Streptocarpus teitensis: implications for conservation of a critically endangered African violet species in Kenya
Source: PeerJ. 2021 Jan 26;9:e10473. doi: 10.7717/peerj.10473 (PMC7845525; doi:10.7717/peerj.10473)

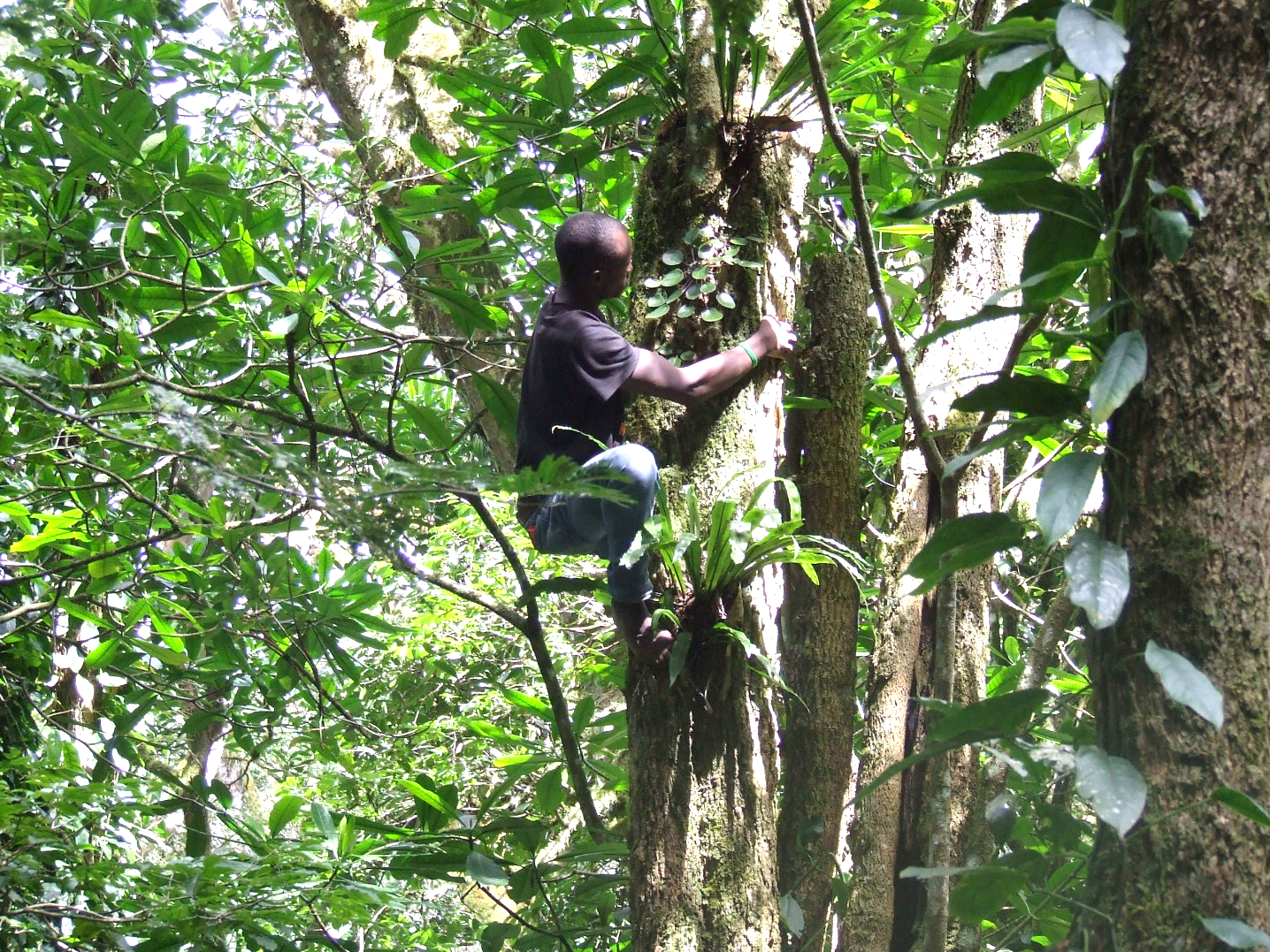

Supplement: Supplemental Information 5 [file peerj-09-10473-s005.png]

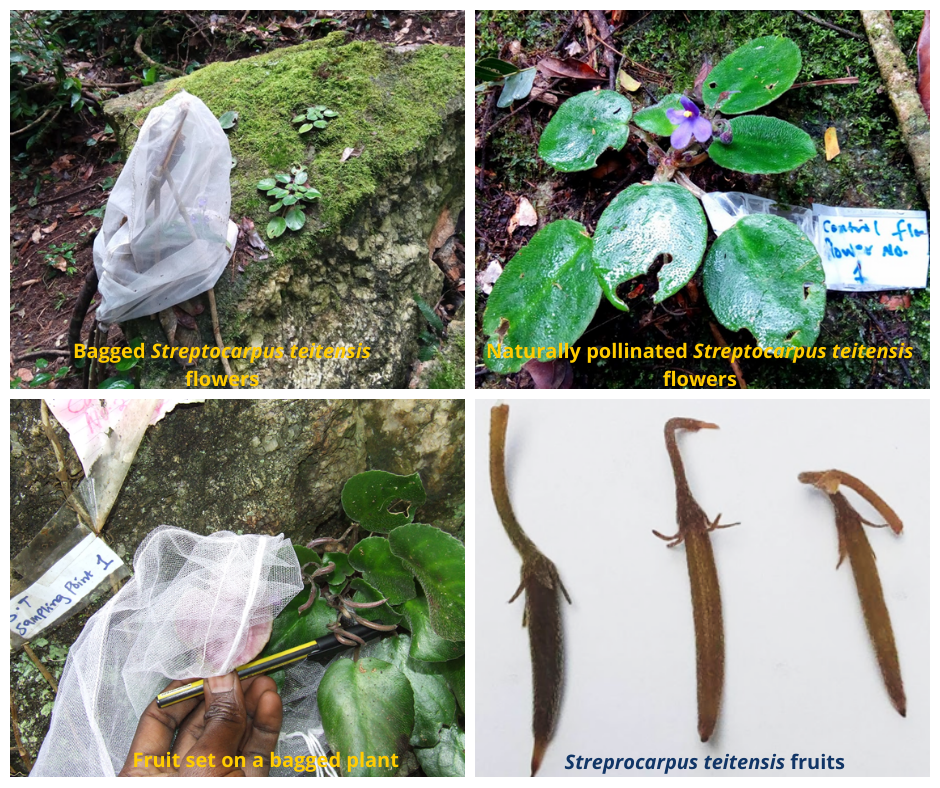

Supplement: Supplemental Information 6 [file peerj-09-10473-s006.png]
