# Supplementary material for: Flower visitors of Streptocarpus teitensis: implications for conservation of a critically endangered African violet species in Kenya
Source: PeerJ. 2021 Jan 26;9:e10473. doi: 10.7717/peerj.10473 (PMC7845525; doi:10.7717/peerj.10473)
Supplement: Supplemental Information 9 — Species strengths of (a) S. teitensis flower visitors visiting other flowering plants and (b) top four S. teitensis flower visitors visiting other flowering plants on diverse dates between 27th April and 2nd August 2019 in Mbololo Forest, Kenya. [file peerj-09-10473-s009.pdf]

Sup 5 (a): Species strength of *S. teitensis* pollinators visiting other wild flowers

S/N	Species	Species strength
1.	*Anthophora conspicua*	6.88
2.	*Anthophora piligera*	0.50
3.	*Anthophora torrida*	2.04
4.	*Apis mellifera*	0.58
5.	*Cathimeris sp*	0.36
6.	*Ceratina sp*	0.75
7.	*Congomochtherus sp*	0.92
8.	*Eumenes sp*	0.36
9.	*Geron sp*	0.45
10.	*Helina coniformis*	0.89
11.	*Liris sp*	0.61
12.	*Megachile cincta*	0.68
13.	*Megachile felina*	0.50
14.	*Megachile sp*	0.58
15.	*Phytomia incisa*	0.58
16.	*Rhingia trivittata*	1.62
17.	*Synagris analis*	1.65
18.	*Xylocopa flavorufa*	0.75
19.	*Xylocopa hottentota*	1.08
20.	*Xylocopa nigrita*	0.20

Sup 5 (b): Species strength of top four *S. teitensis* pollinators visiting other wild flowers

S/N	Species	Species strength
1.	*Anthophora conspicua*	10.07
2.	*Anthophora torrida*	3.12
3.	*Rhingia trivittata*	3.7
4.	*Synagris analis*	3.12
